# Late-Onset Immune-Related Adverse Events After Immune Checkpoint Inhibitor Therapy

**DOI:** 10.1001/jamanetworkopen.2025.2668

**Published:** 2025-03-27

**Authors:** Sienna M. Durbin, Leyre Zubiri, Katherine Perlman, Chia-Yun Wu, Tristan Lim, Kelley Grealish, Nora Hathaway, Jaclyn LoPiccolo, Mike Wang, Ayo Falade, Gabriel Molina, Ted Victor Jacoby, Nishi Shah, Meghan J. Mooradian, Kerry L. Reynolds

**Affiliations:** 1 Division of Hematology & Oncology, Department of Medicine, Massachusetts General Hospital, Boston; 2 Harvard Medical School, Boston, Massachusetts; 3 Department of Dermatology, Massachusetts General Hospital & Harvard Medical School, Boston; 4 Department of Medicine, University of Chicago, Chicago, Illinois; 5 Far Eastern Memorial Hospital, New Taipei City, Taiwan; 6 Dana Farber Cancer Institute, Boston, Massachusetts; 7 Department of Oncology, Mayo Clinic, Rochester, Minnesota; 8 Department of Internal Medicine, Mass General Brigham Salem Hospital, Salem, Massachusetts

## Abstract

**Importance:**

The use of immune checkpoint inhibitors (ICIs) is increasing. Little is known about the frequency of late-onset immune-related adverse events (irAEs) and the patient-specific risk factors associated with their development.

**Objectives:**

To assess the incidence of persistent or de novo late-onset irAEs requiring hospitalization and identify patient factors associated with risk of late-onset irAEs.

**Design, Setting, and Participants:**

This retrospective observational cohort study conducted from January 2011 to October 2022 included patients who received ICIs and were hospitalized with irAEs at an academic medical center. Exclusion criteria included ICI therapy outside of the hospital system and no irAE diagnosis during admission. Data were analyzed from November 15, 2022, to January 8, 2025.

**Exposure:**

Late-onset irAEs.

**Main Outcomes and Measures:**

The main study outcomes were (1) incidence of irAE hospitalization at 0 to 6 months (early), more than 6 to 12 months (intermediate), and more than 12 months (late) after ICI initiation and (2) patient factors associated with risk of late-onset irAEs.

**Results:**

Among the 795 patients hospitalized with irAEs, the median age was 67.3 years (IQR, 58.3-74.8 years); 476 (59.9%) were male. Most patients (n = 517 [65.0%]) received anti–programmed death ligand 1 (PD-L1) and anti–programmed cell death 1 monotherapy, with the most common indications being melanoma (n = 335 [42.1%]) and lung cancer (n = 167 [21.0%]). The median time from start of ICI therapy to hospital admission was 2.7 months (IQR, 1.2-6.1 months), with 14.7% of patients (n = 117 of 795) presenting 6 to 12 months after initial ICI exposure and 10.8% of patients (86 of 795) presenting more than 12 months after initial exposure. The irAEs most likely to present late included those involving the kidney (10 of 32 [31.3%]) and hematologic (5 of 23 [21.7%]) organ systems. In univariate analysis, ICI type was significantly associated with the timing of hospital admission for irAEs; of the 517 patients receiving anti–PD-L1–based therapy, 13.5% (n = 70) presented late compared with 5.4% (9 of 167) receiving dual therapy with anti–cytotoxic T-lymphocyte–associated protein 4 (*P* < .001). Patients receiving perioperative ICI therapy were significantly more likely to be admitted at the intermediate interval (16 of 68 [23.5%]) compared with those with metastatic disease (87 of 678 [12.8%]) (*P* = .03). Timing of irAE was also significantly associated with active ICI exposure; among the patients presenting late, 7.4% (48 of 651) had received ICI therapy within the last 60 days compared with 26.4% (38 of 144) who had not had recent ICI exposure (*P* < .001).

**Conclusions and Relevance:**

The findings of this retrospective observational cohort study suggest that late irAEs are possible, with a subset of patients presenting years after the start of ICI therapy. Clinicians must remain vigilant for irAEs regardless of elapsed time from ICI therapy, especially as patients live longer and ICIs become more widely used. Future investigations are needed to better understand the risk factors for late-onset irAEs and the distinct immunologic pathways that underlie such events.

## Key Points

**Question:**

What is the frequency of late-onset immune-related adverse events (irAEs) among patients treated with immune checkpoint inhibitors (ICIs)?

**Findings:**

In this cohort study of 795 patients, we found that 14.7% of patients hospitalized with irAEs presented within 6 to 12 months after initial ICI exposure and that 10.8% presented more than 1 year after exposure. The irAEs most likely to present late included those involving the kidney (31.3%) and hematologic (21.7%) organ systems.

**Meaning:**

These findings suggest that late irAEs are possible, with a subset admitted for irAE management years after ICI start, underscoring the need for ongoing health care professional vigilance for potential irAEs regardless of elapsed time from ICI therapy.

## Teaser

This cohort study assesses the incidence of persistent or de novo late-onset immune-related adverse events (irAEs) requiring hospitalization and identifies patient factors associated with risk of late-onset irAEs.

## Introduction

Immune checkpoint inhibitors (ICIs) have revolutionized oncology, with approvals across tumor types and clinical trials anticipated to further expand use.^1,2^ Notably, ICIs are now used in adjuvant and neoadjuvant settings, including for melanoma and breast, lung, genitourinary, and esophageal cancers.^3,4,5,6,7,8^ Importantly, although these therapies can be administered with curative intent, with potential for extended survival among patients with early- or advanced-stage disease, clinical trial follow-up periods vary widely based on trial design as well as on type and stage of cancer being studied. The median adverse event (AE) reporting window for ICI clinical trials is only 90 days, and safety data are not always collected for participants who have completed treatment.^9,10^ Thus, there is limited data regarding AEs that may occur years after the start of ICI therapy.

Current literature indicates that most immune-related AEs (irAEs) occur early, typically within the first 6 months of treatment.^11,12^ Myocarditis and endocrinopathies are thought to occur particularly early, with a median time to onset of 4 to 6 weeks, whereas pulmonary and rheumatologic irAEs may present later, with a median time to onset of 34 and 38 weeks, respectively.^11,12,13^ However, the time to irAEs is known to range significantly; for example, guidelines state that colitis may occur between 1 and 107 weeks after treatment initiation and is known to recur after treatment cessation.^12^ Limited data suggest that late-occurring irAEs are possible, diagnosed even years after ICI exposure, but little is known about the frequency of and risk factors for these events.^9,14,15,16,17^

In this study, we aimed to assess the incidence of persistent or de novo late-onset irAEs requiring hospitalization. Specifically, we assessed the occurrence of irAEs within a hospitalized population at distinct temporal intervals (ie, 0-6 months, >6-12 months, and >12 months after ICI initiation). Additionally, we identified patient factors associated with risk of late-onset irAEs. We hypothesized that the risk of irAEs requiring hospitalization would persist beyond the initial months of ICI therapy. By elucidating the frequencies of late-onset irAEs requiring hospital admission within a large, diverse patient population, our intent was to highlight the need for clinicans to remain vigilant regarding the potential diagnosis of irAEs irrespective of the duration elapsed since the commencement of ICI treatment, as well as the need for comprehensive, multidisciplinary care to best serve this complex population.

## Methods

### Study Design

In this retrospective observational cohort study, we included all patients with cancer who received at least 1 dose of any ICI, including anti–programmed cell death 1, anti–programmed death ligand 1 (anti–PD-L1), and anti–cytotoxic T-lymphocyte–associated protein 4 (anti–CTLA-4) as monotherapy or in combination with other antineoplastics, and were subsequently hospitalized at Massachusetts General Hospital between January 2011 and October 2022. Participants were identified using pharmacy and hospital admission databases. On October 3, 2017, the Mass General Cancer Center established the Severe Immunotherapy Complications service, staffed by expert oncologists with subspecialty input. Before 2017, these patients were identified using electronic health record documentation. After October 2017, this group prospectively screened all patients with ICI exposure on admission to the hospital. All cases were reviewed by experts, who categorized irAE diagnoses into “confirmed” via characteristic biopsy or imaging findings; “suspected” by ruling out other possible causes; and “not toxicity.” Cases were reviewed according to the previously published methods for this database.^18^ Because clinical severity was defined by whether the irAE required hospitalization, Common Terminology Criteria for Adverse Events grading was not recorded, although, by definition, nearly all irAEs requiring admission are considered serious (grade ≥3).^19^ Patients without a suspected or confirmed irAE diagnosis were excluded, as were those who received ICI therapy outside of our hospital system. The study protocol was approved by the Mass General Brigham institutional review board. The reporting of results adheres to the Strengthening the Reporting of Observational Studies in Epidemiology (STROBE) reporting guideline, ensuring methodological transparency and rigor. Given the retrospective design, a waiver of informed consent was obtained from the institutional review board because the study used deidentified data and posed minimal risk to participants. This approach aligns with the ethical standards and regulatory requirements for human participant research.

### Demographic and Clinical Characteristics

We extracted data from the electronic health record regarding patients’ age, sex, and cancer type. We extracted information regarding the dates of all ICI exposures and the type of the most recent ICI exposure (grouped into anti–PD-L1, anti–CTLA-4, and anti–PD-L1/CTLA-4 combination), as well as exposure to steroids and steroid-sparing secondary immunosuppression. We collected data on the indications for an ICI, categorized into treatment for metastatic or stage IV disease, perioperative therapy (defined as neoadjuvant therapy with or without adjuvant therapy), and treatment for locally unresectable disease, from the documentation of the treating oncologist. Patients who had received an ICI within 60 days of admission were categorized as receiving active therapy. Data were censored on October 15, 2022.

### irAE Categorization

Using the date of the first dose of an ICI (cycle 1, day 1) received closest to hospital admission, we calculated the time from the start of ICI therapy to hospital admission. We then examined the number of irAE hospitalizations between 0 and 6 months after the start of ICI therapy (defined as early-onset irAEs), more than 6 to 12 months (defined as intermediate-onset irAEs), and more than 12 months (defined as late-onset irAEs).

### Statistical Analysis

We used descriptive statistics, including analyses of variance and χ^2^ tests, to evaluate univariate associations between patient characteristics and time of irAE presentation. We also examined the median time to presentation for irAEs. Two-sided *P* < .05 indicated a statistically significant difference. The statistical analysis was performed from November 15, 2022, to January 8, 2025, using Excel software, version 16.94 (Microsoft).

## Results

During the study period, there were a total of 5236 patients treated with an ICI-containing therapy. We included a total of 795 patient admissions, excluding those who received their ICI therapy outside of our medical system and those who, determined by expert review, did not have an irAE diagnosed during admission. In this cohort, the median age was 67.3 years (range, 18.3-96.2 years), and most patients (n = 476 [59.9%]) were male. The most common tumor types included melanoma (n = 335 [42.1%]) and lung cancer (n = 167 [21.0%]). Most patients were treated with anti–PD-L1–based therapy (n = 517 [65.0%]) in the metastatic setting (n = 678 [85.3%]), and most patients (n = 651 [81.9%]) received active therapy. Some patients (n = 46 of 795 [5.8%]) died during hospital admission; of 749 patients discharged from the hospital, 13.8% (n = 103) were readmitted for the same irAE. Most patients were discharged and were taking steroids (n = 600 [80.1%]), and a subset of patients required additional immunosuppression (56 of 795 [7.0%]). Of those patients not discharged or taking steroids, one-third had either endocrine toxic effect (n = 30 [20.1%]) or dermatologic toxic effect (n = 15 [10.1%]). The median time from the start of ICI therapy to hospital admission was 2.7 months (IQR, 1.2-6.1 months), with 117 patients (14.7%) presenting 6 to 12 months after initial ICI exposure and 86 patients (10.8%) presenting more than 12 months. Among those patients presenting with a late toxic reaction, 77.9% (n = 67) were receiving first-line therapy, with most patients (n = 70 [81.4%]) treated with PD-L1 inhibitors and only 10.5% (n = 9) receiving combination therapy. Additionally, although most patients with late irAEs (n = 75 [87.2%]) had metastatic disease, some (n = 4 [4.7%]) were treated in the perioperative setting for management of earlier-stage disease (Table 1). Patients were followed up for a median of 24.3 months (IQR, 8.01-56.1 months) from the time of ICI exposure to the date of last follow-up.

**Table 1.** Univariable Associations Between Patient Characteristics and Time of Hospital Admission for irAEs

Characteristic	Patients, No. (%)				*P* value
	All	With early irAEs (0-6 mo)	With intermediate irAEs (>6-12 mo)	With late irAEs (>12 mo)	
No.	795	592	117	86	
Age, median (IQR), y	67.3 (58.3-74.8)	66.9 (58.1-74.5)	68.9 (61.5-76.1)	65.9 (58.2-75.7)	.04
Sex					
Male	476 (59.9)	357 (75.0)	69 (14.5)	50 (10.5)	.91
Female	319 (40.1)	235 (73.7)	48 (15.0)	36 (11.3)	
Cancer type					
Melanoma	335 (42.1)	266 (79.4)	42 (12.5)	27 (8.1)	.003
Lung	167 (21.0)	116 (69.5)	30 (18.0)	21 (12.6)	
Gastrointestinal	83 (10.4)	65 (78.3)	14 (16.9)	4 (4.8)	
Genitourinary	65 (8.2)	42 (64.6)	9 (13.8)	14 (21.5)	
Head and neck	40 (5.0)	27 (67.5)	7 (17.5)	6 (15.0)	
Gynecologic	35 (4.4)	21 (60.0)	4 (11.4)	10 (28.6)	
Breast	31 (3.9)	23 (74.2)	5 (16.1)	3 (9.7)	
Other^a^	39 (4.9)	32 (82.1)	6 (15.4)	1 (2.6)	
Prior lines of ICI					
None	647 (81.4)	480 (74.2)	100 (15.5)	67 (10.4)	.37
≥1	148 (18.6)	112 (75.7)	17 (11.5)	19 (12.8)	
Most recent ICI					
Anti–PD-L1	517 (65.0)	346 (66.9)	101 (19.5)	70 (13.5)	<.001
Anti–PD-L1/CTLA-4 combination	167 (21.0)	149 (89.2)	9 (5.4)	9 (5.4)	
Anti–CTLA-4	111 (13.0)	97 (87.4)	7 (6.3)	7 (6.3)	
Indication for ICI^b^					
Metastatic	678 (85.3)	516 (76.1)	87 (12.8)	75 (11.1)	.03
(Neo)adjuvant	68 (8.6)	48 (70.6)	16 (23.5)	4 (5.9)	
Locally unresectable	49 (6.2)	28 (57.1)	14 (28.6)	7 (6.3)	
Received ICI within 60 d of admission					
Yes	651 (81.9)	534 (82.0)	69 (10.6)	48 (7.4)	<.001
No	144 (18.1)	58 (40.3)	48 (33.3)	38 (26.4)	
Multiple irAEs during admission					
Yes	89 (11.2)	73 (82.0)	11 (12.4)	5 (5.6)	.16
No	706 (88.8)	519 (73.5)	106 (15.0)	81 (11.5)	
Died during admission					
Yes	46 (5.8)	37 (80.4)	4 (8.7)	5 (10.9)	.49
No	749 (94.2)	555 (74.1)	113 (15.1)	81 (10.8)	
Discharged receiving steroids^c^					
Yes	600 (80.1)	450 (75.0)	88 (14.7)	62 (10.3)	.49
No	149 (19.9)	105 (70.5)	25 (16.8)	19 (12.8)	
Treated with secondary immunosuppression					
Yes	56 (7.0)	42 (75.0)	9 (16.1)	5 (8.9)	.87
No	739 (93.0)	550 (74.4)	108 (14.6)	81 (11.0)	
Received ICI after discharge^c^^,^^d^					
Yes	203 (33.1)	178 (87.7)	15 (7.4)	10 (4.9)	<.001
No	410 (66.9)	321 (78.3)	52 (12.7)	37 (9.0)	
Readmission for same irAE^c^					
Yes	103 (13.8)	78 (75.5)	12 (11.7)	13 (12.6)	.49
No	646 (86.2)	477 (73.8)	101 (15.6)	68 (10.5)	

Abbreviations: CTLA-4, cytotoxic T-lymphocyte–associated protein 4; ICI, immune checkpoint inhibitor; irAEs, immune-related adverse events; PD-L1, programmed death ligand 1.

^a^ Includes primary central nervous system cancers (n = 20), hematologic cancers (n = 17), and sarcoma (n = 2).

^b^ Statistical analysis excludes locally unresectable given heterogeneity of group.

^c^ Excludes those who died during admission.

^d^ Excludes those who had not received an ICI within 60 days of admission.

In univariate analysis, cancer type was significantly associated with timing of irAE diagnosis; for example, of 335 patients who received a diagnosis of melanoma, 79.4% (n = 266) presented early and 8.1% (n = 27) presented late. This is in contrast to lung cancer, where 69.5% of patients (116 of 167) presented early and 12.6% of patients (21 of 167) presented late (*P* = .003). Age was significantly associated with irAE timing, with a median age of 66.9 years (IQR, 58.1-74.5 years) admitted early, 68.9 years (IQR, 61.5-76.1 years) admitted intermediately, and 65.9 years admitted late (IQR, 58.2-75.7 years) (*P* = .04). We also identified an association between ICI type and irAE timing; of 517 patients treated with anti–PD-L1–based therapy, 66.9% (n = 346) presented with early-onset, 19.5% (n = 101) presented with intermediate-onset, and 13.5% (n = 70) presented with late-onset irAEs. Patients with anti–CTLA-4 exposure were less likely to present late, with 5.4% (9 of 167) of those treated with combination checkpoint blockade and 6.3% (7 of 111) of those treated with CTLA-4 monotherapy presenting late (*P* < .001). Patients receiving perioperative ICI were significantly more likely to be admitted at the intermediate interval (16 of 68 [23.5%]) compared with those with metastatic disease (87 of 678 [12.8%]) (*P* = .03). There was no significant difference in the risk of multiple irAE diagnoses between patient groups, although there was a trend toward increased admission of multiple irAEs in the early group (73 of 89 [82.0%] with multiple irAEs vs 519 of 706 [73.5%] without; *P* = .16). Finally, the timing of irAEs was significantly associated with active ICI exposure. Of 651 patients who received active treatment, 82.0% (n = 534) presented with early-onset, 10.6% (n = 69) presented with intermediate-onset, and 7.4% (n = 48) presented with late-onset irAEs compared with 144 patients not receiving active treatment, within which 40.3% (n = 58) presented with early-onset, 33.3% (n = 48) presented with intermediate-onset, and 26.4% (n = 38) presented with late-onset irAEs (*P* < .001).

The median time from the most recent dose of an ICI to hospital admission was 0.7 months (IQR, 0.4-1.5 months), with 27 of 795 patients (3.4%) presenting more than 6 months after the last dose of an ICI and 5 of 795 patients (0.6%) presenting more than 1 year after the last ICI exposure. Among those patients receiving active therapy, the timing of presentation was significantly associated with the likelihood of ICI rechallenge after discharge; of 203 patients rechallenged, 87.7% (n = 178) presented with early-onset, 7.4% (n = 15) presented with intermediate-onset, and 4.9% (n = 10) presented with late-onset irAEs, whereas the 410 patients not rechallenged were less likely to present with early-onset irAEs (78.3% [n = 321]) and more likely to present with late-onset irAEs (9.0% [n = 37]) (*P* < .001). There was no significant difference in the risk of death during hospital admission; for the 86 patients presenting with late-onset irAEs, 10.9% ( 5 of 46 patients with multiple irAEs) died during hospital admission compared with 10.8% (81 of 749 patients with multiple irAEs) who did not (*P* = .49). The most common irAE diagnoses for patients who died during admission included the pulmonary (39.1% [n = 18]) and cardiac (17.4% [n = 8]) organ systems. Of the 46 patients who presented late with fatal irAEs, diagnoses included the gastrointestinal (2 of 5 [40%]), endocrine (1 of 5 [20%]), hepatic (1 of 5 [20%]), and pulmonary (n = 1 [2.2%]) organ systems.

Overall, regardless of timing, the most frequently occurring irAEs included gastrointestinal (233 of 898 [25.9% of all irAEs]) and pulmonary (128 of 898 [14.3%]). The irAEs that were most likely, proportionally, to be associated with late hospital admission included those involving the kidney (10 of 32 [31.3% of all kidney cases presented late]), hematologic (5 of 23 [21.7%]), and rheumatologic (4 of 26 [15.4%]) organ systems. Notably, although some irAEs were more common in the early and intermediate stages, all diagnoses were seen throughout. For example, 82.8% (53 of 64) of all cardiac irAEs were early, but 6.3% (4 of 64) were seen late; neurologic irAEs were also often seen early (81.4% [70 of 86]), although 4.7% (4 of 86) were late (Table 2). The irAE with the earliest median time to onset was cardiac (2.12 months [IQR, 1.13-5.07 months]). Kidney irAEs had the longest median time to onset (4.83 months [IQR, 2.07-14.0 months]) (Figure).

**Table 2.** Immune-Related Adverse Event Diagnoses by Time of Admission

Organ system	Events, No. (%)				
	All	Early (0-6 mo)	Intermediate (>6-12 mo)	Late (>12 mo)
No.	898	679	128	91
Gastrointestinal	233 (25.9)	183 (78.5)	29 (12.4)	21 (9.0)
Pulmonary	128 (14.3)	91 (71.1)	21 (16.4)	16 (12.5)
Hepatic	120 (13.4)	96 (80.0)	14 (11.7)	10 (8.3)
Endocrine	115 (12.8)	91 (79.1)	17 (14.8)	7 (6.1)
Neurologic	86 (9.6)	70 (81.4)	12 (13.9)	4 (4.7)
Cardiac	64 (7.1)	53 (82.8)	7 (10.9)	4 (6.3)
Dermatologic	53 (5.9)	36 (67.9)	10 (18.9)	7 (13.2)
Kidney	32 (3.6)	18 (56.2)	4 (12.5)	10 (31.3)
Rheumatologic	26 (2.9)	17 (65.4)	5 (19.2)	4 (15.4)
Hematologic	23 (2.6)	12 (52.2)	6 (26.1)	5 (21.7)
Other^a^	18 (2.0)	12 (66.7)	3 (16.7)	3 (16.7)

^a^ Includes fever (n = 8), allergy (n = 7), ocular event (n = 2), and fatigue (n = 1).

**Figure.**
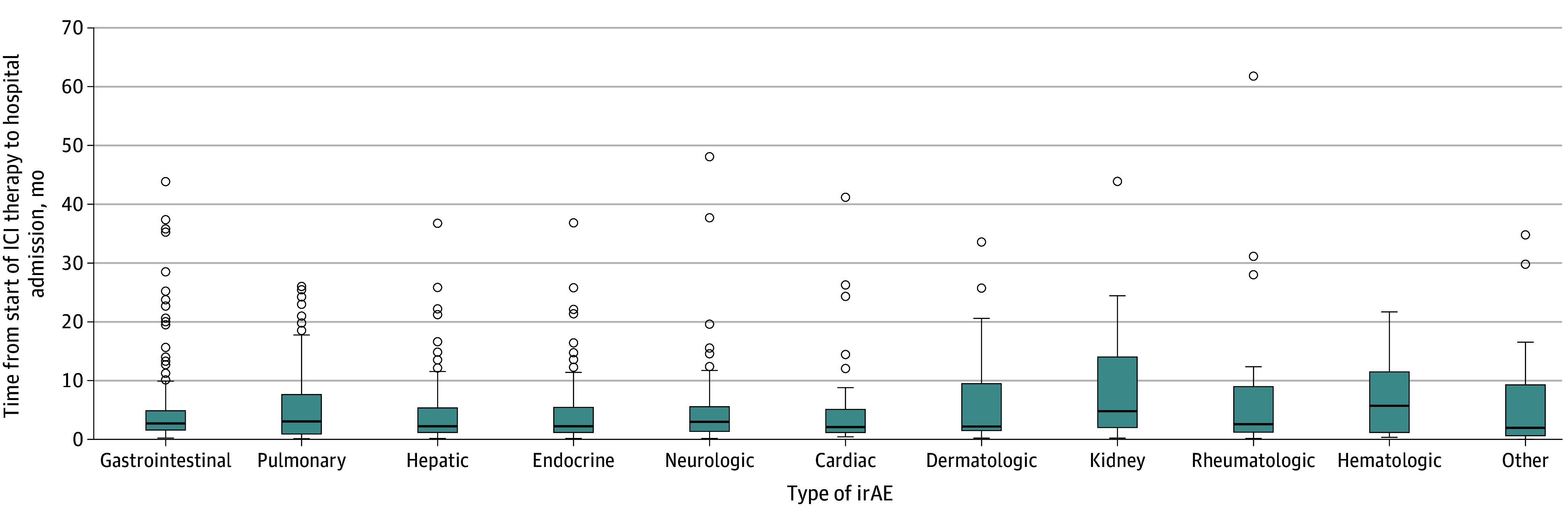
Box Plot of Time From Start of Immune Checkpoint Inhibitor (ICI) to Hospital Admission by Diagnosis of Immune-Related Adverse Event (irAE) Within each box, the horizontal line denotes median values. Boxes extend from the 25th to 75th percentile, with vertical lines extending to the most extreme values within the 1.5 IQR of the 25th and 75th percentile. Dots indicate outlier observations. “Other” includes primary central nervous system cancers (n = 20), hematologic cancers (n = 17), and sarcoma (n = 2).

## Discussion

In this retrospective cohort study, which represents a large database of hospitalized patients with irAEs, we observed that although most irAEs occur within the first 6 months of therapy, delayed onset of toxic effects can occur, with the risk of irAEs requiring hospitalization persisting for more than 5 years after initial ICI exposure. We identified patient characteristics associated with the timing of irAEs and found significant associations between late-onset irAEs and cancer type and between type and indication of ICI. We also found that patients admitted to the hospital with late-onset irAEs are less likely to be receiving active therapy and less likely to receive a further ICI after hospital discharge. We identified certain irAEs, including those in the kidney and hematologic organ systems, that exhibit a higher propensity to manifest later in the course of ICI therapy. Collectively, these findings highlight the need for vigilance for irAEs at any point both during and after a patient’s treatment with an ICI and establish hypothesis-generating associations of ICI type and indication with the risk of late-onset irAEs.

To our knowledge, this study represents the first large-scale effort to quantify the incidence of late irAEs in a heterogeneous group of patients treated with ICIs. We identified that patients experiencing late toxic effects predominantly had melanoma, followed by lung and genitourinary cancers. Among the 86 patients who encountered 91 late toxic effects, most were receiving first-line therapy with PD-L1 inhibitors and had metastatic disease, although a few were treated in the perioperative setting for earlier-stage disease. Notably, we found that multiple readily available demographic factors, including sex and prior exposure to an ICI, have no significant association with increased risk of late hospital admission. We note 1 caveat: although age met statistical significance in univariable analysis, the clinical relevance of this finding is uncertain because the absolute difference in age between groups was minimal. Our findings do suggest that patients with certain tumor types, including lung, genitourinary, and gynecologic cancers, may have a relatively higher risk of late presentation; the underlying mechanism of this pattern is unclear, and further work is needed to better characterize the potential associations between these patient populations and the treatments they received with the risk of late-occurring irAEs. Potential hypotheses for mechanisms of late-occurring irAEs include heterogeneity in the tumor microenvironment^20^ and variable duration of ICI therapy, as well as exposure to subsequent therapies, such as targeted therapies, that may increase the risk of the development of irAEs.^21,22^ Future work is also needed to identify host factors, including gene polymorphisms in immune-related genes, that may increase the risk of late-occurring irAEs.^23^ Although not meeting statistical significance, we also identified a trend toward decreasing presentation of multiple irAEs in the late population. Overall, our exploratory work highlights the need for multi-institutional collaboration to pool cases for optimal analysis to better understand the interplay between tumor biology, treatment context, and patient-specific factors to identify at-risk populations for delayed irAEs.

In this analysis, patients treated with anti–PD-L1–based therapies were more likely to present later compared with those receiving combination ICI therapy. These findings had been previously described in a cohort of patients with melanoma and lung cancer presenting with delayed irAEs, with delayed presentation more common among those treated with anti–PD-L1 monotherapies, and were posited to be explained by longer treatment duration.^17^ It has been well demonstrated in clinical trials that dual ICI therapy increases the risk of irAEs, particularly severe, earlier-onset toxic effects.^24,25^ In our data, this does not appear to translate to an increased risk of late-onset irAEs requiring hospital admission, perhaps because of the finite course of CTLA-4 inhibition used in most treatment regimens.^24^ There was also a significant association between treatment indication and irAE timing, with those treated with perioperative ICIs more likely to present with intermediate-onset irAEs and less likely to present with late-onset irAEs compared with those with metastatic disease. We hypothesize that this may be associated with the duration of ICI therapy in the treatment of early-stage disease, which is often a maximum of 1 year,^5,8,26^ whereas the optimal duration of ICI therapy for advanced disease is unknown, with data suggesting some patients receive therapy for more than 2 years.^27^ Finally, we found that nearly half of late presentations occurred after ICI therapy had been stopped. Importantly, we demonstrate that patients can present at any time with toxic effects, and thus suspicion must remain high for the development of irAEs even after receipt of ICI therapy. This is particularly important because the timing between clinical visits can be prolonged after patients transition from active therapy to surveillance, which may lead to delayed diagnoses of irAEs and the potential for increased toxic effects. Therefore, letting patients know that the development of irAEs remains possible even after ICI cessation is paramount.

Although we demonstrated that kidney, hematologic, and rheumatologic irAEs may be more likely to occur late, we also show that all organ systems can be affected by late-onset irAEs. Cardiac and neurologic toxic effects are particularly morbid, accounting for nearly one-third of all deaths attributable to irAEs in 1 analysis^28^ and, in our dataset, accounted for 7.1% (64 of 898) and 9.6% (86 of 898) of hospital admissions, respectively. Although most of these cases were seen in the first 6 months, our data demonstrate that late-onset myocarditis and neurologic toxic effects, although uncommon, are possible. These findings are in line with previously published case reports, which highlight the potential for late-onset, ICI-associated myocarditis.^29,30^ Although we did not identify any cases of fatal late-onset cardiac or neurologic toxic effects in this dataset, we demonstrate no significant difference in the risk of fatal toxic effects across early, intermediate, or late presentations, suggesting that there remains a risk of severe irAEs even years after ICI exposure. There is also no difference in the need for secondary immunosuppression, suggesting that late-presenting grade 5 irAEs may be similarly refractory to steroids when compared with early presentations. Building on the need for vigilance, we also highlight that hospitalization for multiple irAEs was possible more than 1 year from the start of ICI therapy, demonstrating that multisystem involvement must remain on the differential for patients with prior ICI exposure. More work is needed to understand why certain organ systems may be more vulnerable to late toxic effects and to assess whether these late diagnoses represent a distinct irAE phenotype.

### Limitations

Limitations to our study include its retrospective, single-institution design; these results may be less generalizable to other settings. Our Severe Immunotherapy Complications service provides novel multidisciplinary care to patients treated with ICIs, which may lead to unique results when compared with institutions without structured care for patients with irAEs.^31^ With our center’s expertise, we may also demonstrate higher rates of ICI rechallenge and lower rates of readmission than may be seen in other clinical practices. Additionally, although the expertise of the Severe Immunotherapy Complications service helps to identify irAEs, with the increasing use of therapies combining ICIs with other neoplastic agents, the ability to fully adjudicate an irAE can be challenging. Certain data points, such as grade of toxicity and cancer-specific outcomes, were also lacking. This prevents rigorous comment on the severity of adverse events as well as decisions regarding ICI rechallenge based on tumor response. Furthermore, the Severe Immunotherapy Complications service bridges the inpatient and outpatient settings and allows for many admissions to be avoided via urgent irAE clinics. As such, these data, which are limited to the inpatient setting, may underestimate rates of clinically significant irAEs that are managed in an outpatient setting. We also lack information regarding preexisting outpatient diagnoses. Although we present the exact date that irAEs require inpatient management, this may not reflect the exact date of irAE development. It is possible that some presentations may represent a new complication or recurrence of a previously diagnosed irAE that was managed in an outpatient setting. Our dataset was not adequately powered for multivariable analysis. As previously discussed, multi-institutional collaborations are necessary to create a cohort of late-presenting irAE diagnoses to further investigate the hypotheses posited in this work.

## Conclusions

In this retrospective observational cohort study, our findings demonstrated the heterogeneity among individuals with irAEs requiring hospitalization. We showed that, although most irAEs occur early within the course of ICI therapy, late-onset serious toxic effects requiring hospitalization were possible across all organ systems. As ICIs are used across a diversity of cancer types, especially in early-stage disease, the likelihood increases that clinicians will encounter patients surviving for extended periods after ICI exposure. Our data highlighted that all clinicians within the health care system must remain vigilant for irAEs regardless of elapsed time from receipt of ICIs. Future investigation is needed to better understand the risk factors for delayed irAEs and the distinct immunologic pathways that may underlie such events.

## Notes

Supplement.
Data Sharing Statement
