# Supplementary material for: Late-Onset Immune-Related Adverse Events After Immune Checkpoint Inhibitor Therapy
Source: JAMA Netw Open. 2025 Mar 27;8(3):e252668. doi: 10.1001/jamanetworkopen.2025.2668 (PMC11950896; doi:10.1001/jamanetworkopen.2025.2668)
Supplement: Supplement. — Data Sharing Statement [file jamanetwopen-e252668-s001.pdf]

Data Sharing Statement

Durbin. Late-Onset Immune-Related Adverse Events After Immune Checkpoint Inhibitor Therapy. *JAMA Netw Open*. Published March 27, 2025.
doi:10.1001/jamanetworkopen.2025.2668

Data
Data available: No
